# National Dissemination of Chronic Disease Self-Management Education Programs: An Incremental Examination of Delivery Characteristics

**DOI:** 10.3389/fpubh.2014.00227

**Published:** 2015-04-27

**Authors:** Matthew Lee Smith, Marcia G. Ory, SangNam Ahn, Kristie P. Kulinski, Luohua Jiang, Scott Horel, Kate Lorig

**Affiliations:** ^1^Department of Health Promotion and Behavior, College of Public Health, The University of Georgia, Athens, GA, USA; ^2^Department of Health Promotion and Community Health Sciences, Texas A&M Health Science Center, School of Public Health, College Station, TX, USA; ^3^Division of Health Systems Management and Policy, School of Public Health, The University of Memphis, Memphis, TN, USA; ^4^National Council on Aging, Washington, DC, USA; ^5^Department of Epidemiology and Biostatistics, Texas A&M Health Science Center, School of Public Health, College Station, TX, USA; ^6^Department of Medicine, Stanford Patient Education Research Center, Stanford School of Medicine, Palo Alto, CA, USA

**Keywords:** chronic disease self-management, evidence-based program, older adults, sustainability, program implementation, program reach, evaluation

## Abstract

With a near 20-year developmental history as an evidence-based program, the suite of Chronic Disease Self-Management Education (CDSME) programs were selected in 2010 for grand-scale dissemination in a federally supported initiative to improve the health of older Americans. The primary charge of this national effort was to establish a sustainable program delivery system for empowering American adults with one or more chronic conditions to better manage their health. The current study focused on a series of dissemination and implementation science research questions to: (1) examine the geographic distribution of participation in this initiative across the Unites States; (2) describe workshop characteristics engaged to reach program participants in various settings; and (3) describe personal characteristics of the first 100,000 participants. Each subsequent entering cohort was descriptively examined to indicate whether there was constancy or change in delivery sites and populations reached over time. Findings show a strengthening of the workshop delivery infrastructure in that it took 9.4 months to reach the first 25,000 participants in 853 counties compared to 5.4 months to reach the last 25,000 participants in 1,109 counties. The workshop delivery characteristics and participant characteristics remained relatively consistent across increments of 25,000 participants reached, although general trends were observed for some variables. For example, after reaching the first 25,000 participants, subsequent groups of 25,000 participants were reached more quickly. Additionally, workshops were increasingly delivered in ZIP Codes with higher percentages of families residing below the federal poverty line. As more participants were reached, more participants with chronic conditions were enrolled. This national translational study illustrates the rapid expansion of CDSME programs throughout the United States and capability to reach diverse populations in a variety of settings.

## Introduction

Seen as a critical part of primary care for the past 20 years (1, 2), disease self-management programs have been associated with a plethora of positive health outcomes among middle-aged and older adults in the United States (3). While the healthcare system is increasingly expected to provide chronic care (1), chronic disease self-management initiatives outside of the physician’s office are now widely recognized as an effective complement to improve health indicators and quality of life while reducing overall health-related complications and associated costs (4). One of the most extensively tested programs, the Stanford Chronic Disease Self-Management Program (CDSMP), is a 6-week program (5) that has strong evidence demonstrating its ability to improve participants’ health status, modify their health behaviors, and reduce their healthcare utilization and associated costs (6–9). The interactive workshop sessions are designed to enhance three types of skills necessary for the everyday management of chronic conditions: medical management, emotional management, and social role management (6). While CDSMP remains the flagship program, Stanford has translated it to be delivered online, in multiple languages, and for specific diseases/conditions (e.g., diabetes, arthritis, chronic pain, HIV) (5). This collection of interventions (including CDSMP) comprises the suite of Chronic Disease Self-Management Education (CDSME) programs.

Building on a nascent evidence-based prevention initiative supported by the U.S. Administration on Aging (AoA) beginning in 2003 (10), funding was provided as part of the American Recovery and Reinvestment Act of 2009 (ARRA) to disseminate CDSME programs in 45 states, Puerto Rico, and the District of Columbia between 2010 and 2012 (11). Given the solid evidence base behind CDSMP, this jointly administered initiative of the AoA, the Centers for Disease Control and Prevention (CDC), and the Centers for Medicare and Medicaid Services (CMS) sought to bring these evidence-based programs to scale for the important goal of addressing the rapidly rising number of older adults struggling to manage their chronic conditions. The national goal of this ARRA-funded initiative was to reach at least 50,000 program completers (i.e., attend four or more of the six workshops sessions). Each participating state and entity was assigned a target goal for program completers based on the size of its population of older Americans. There was an expectation that certain delivery site types would be utilized (e.g., senior centers, healthcare organizations, residential facilities, educational institutions, faith-based organizations, and tribal centers), and special emphasis was placed on recruiting and enrolling racial/minority and other underserved populations.

The goal of having over 50,000 adults complete CDSME program workshops was accomplished within the first 24 months of this initiative across more than 1,000 United States counties (12). This accomplishment demonstrates the feasibility of a coordinated effort with the aging services network, the public health, and healthcare sectors. This study examined participant accrual of the first 100,000 participants enrolled in this national CDSME program roll out in four blocks (i.e., each representing 25,000 participants). Using this frame of progressing accrual blocks, the purposes of this study were to: (1) examine the geographic distribution of participation in this initiative across the Unites States; (2) describe workshop characteristics engaged to reach program participants in various settings; and (3) describe personal characteristics of the first 100,000 participants. Each subsequent entering cohort was descriptively examined to indicate whether there was constancy or change in delivery sites and populations reached over time.

## Materials and Methods

### Chronic disease self-management education (CDSME) programs

As described previously, CDSMP falls within a suite of CDSME programs that have been widely disseminated in the U.S. as a method to empower patients with self-management skills to deal with their chronic conditions (12, 13). Drawing upon Social Learning Theory (14), CDSMP is an evidence-based, peer-led intervention consisting of six highly participative classes held for 2.5 h each, once a week, for six consecutive weeks (13). Additional details about the theory behind CDSME programs and their implementation can be found elsewhere (15).

### Data source and study population

This study reports findings based on cross-sectional data collected from the first 100,000 participants enrolled in the nationwide delivery of CDSME programs as part of the American Recovery and Reinvestment Act of 2009 (i.e., Recovery Act) *Communities Putting Prevention to Work: Chronic Disease Self-Management Program* initiative (12). Workshops were delivered in 45 states, Puerto Rico, and the District of Columbia (11). With support from AoA, a centralized online data system was developed by the National Council on Aging to collect data from participating organizations (15). Each state identified several database users at the state- and/or regional-level who were responsible for entering workshop and participant data.

### Measures

In recognition of the importance of minimizing assessment burden, the data collection effort was limited to a short informational sheet about the delivery organization to be filled out by the delivery sites; a brief set of items describing participant characteristics such as age, sex, race/ethnicity, number and type of self-reported chronic conditions, living arrangements and ZIP Code (for participant residence and delivery site location); and attendance logs to document the specific sessions attended by each participant. While the expectation was that each organization would collect all the data referenced above, due to privacy and other concerns at some locations, all of the data elements were not collected at all of the sites (15). Further, because the completion of the participant questionnaire was not a pre-requisite for attending the workshop, some delivery sites chose not to collect all data points and some participants elected not to complete the questionnaire. However, to be counted as a “successful” completer (i.e., attending four of the six offered workshop sessions), the workshop information sheet and attendance roster was required.

### Analyses

Statistical analyses for this study were performed using SPSS (version 21). Workshop and participant characteristics were compared between the first, second, third, and fourth group of 25,000 participants reached. Additionally, maps were generated to illustrate the cumulative geospatial distribution and accruement of CDSME program participants and workshops for the first 25,000 participants, 50,000 participants, 75,000 participants, and all 100,000 participants. Plots indicate workshop locations. Shading indicates the number of participants reached in each state (i.e., darker shade represents more participants reached). Hash markings represent the first year in which funding was received by state.

## Results

### National CDSME program uptake

**Figure 1:**
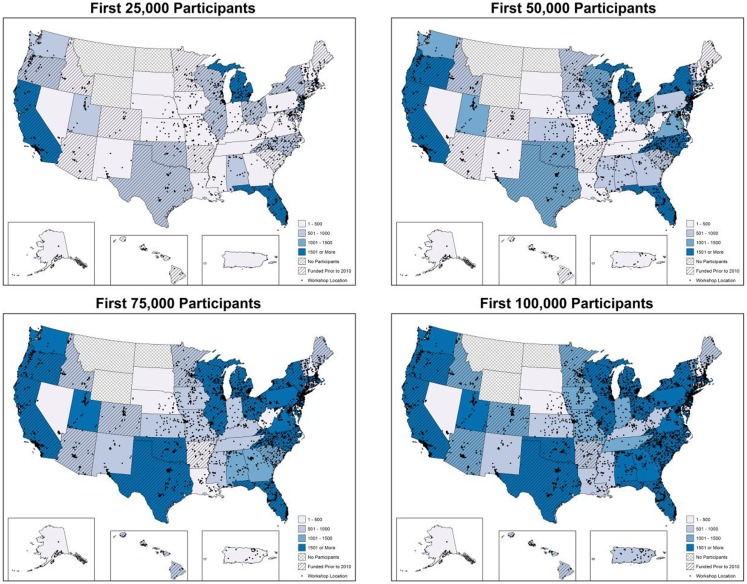
**Geospatial distribution of CDSMP workshops and participants by increments of 25,000 participants**.

Figure 1 depicts the cumulative geospatial distribution of the first 100,000 CDSME program enrollees by increments of 25,000 participants. As can be seen, the first 25,000 participants were reached by 2,226 workshops in 1,705 unique implementation sites over a 9.4-month period across 853 counties. At this stage in the intervention, only a few states had reached over 1,000 participants. Comparatively, the last 25,000 participants were reached by 2,154 workshops in 1,769 unique implementation sites over a 5.4-month period across 1,109 counties. At this stage in the intervention, only a few states had not reached over 1,000 participants.

### CDSME program workshop characteristics

Table 1 presents workshop characteristics for the first 100,000 CDSME program participants enrolled in the intervention. These 100,000 participants were reached by 8,702 workshops in 5,586 unique implementation sites over a 25.9 month period across 1,786 counties. The majority of participants enrolled in CDSMP workshops (78.4%), followed by Diabetes Self-Management Program (DSMP) workshops (10.3%) and Tomando Control de su Salud (Spanish CDSMP) workshops (8.9%). The largest proportion of participants attended workshops at senior centers or area agencies on aging (29.2%), followed by healthcare organizations (21.1%), residential facilities (17.6%), community/multi-purpose facilities (9.9%), faith-based organizations (8.4%), and other settings (e.g., correctional facilities malls, RV parks, fire departments, county administration buildings, private residences, casinos, career centers). The majority of participants attended workshops delivered in English (89.6%) and in metro settings (79.6%). On average, workshops included 12.69 (±4.18) participants, and participants attended 4.38 (±1.72) sessions. The workshop completion rate was 74.9%.

**Table 1:** **Workshop characteristics by reach increments of 25,000 participants**.

	Total	Participant 1–25,000	Participant 25,001–50,000	Participant 50,001–75,000	Participant 75,001–100,000
**Number of unique counties served**	1,786	853	1,048	988	1,109
**Number of workshops delivered**	8,702	2,226	2,138	2,184	2,154
**Number of unique implementation sites**	5,586	1,705	1,727	1,764	1,769
**Time to enroll (in months)**	25.91	9.37	6.00	5.17	5.37
**Participants reached by CDSME workshop type**					
Arthritis self-management program (ASMP)	0.5%	0.8%	0.4%	0.1%	0.5%
Chronic disease self-management program (CDSMP)	78.4%	80.2%	79.5%	75.6%	78.3%
Chronic pain self-management program (CPSMP)	0.5%	0.0%	0.6%	0.4%	1.0%
Diabetes self-management program (DSMP)	10.3%	8.3%	9.4%	11.3%	12.0%
Spanish ASMP	0.1%	0.3%	0.0%	0.0%	0.0%
Tomando control de su dabetes (Spanish DSMP)	1.4%	0.8%	1.1%	2.5%	1.2%
Tomando control de su salud (Spanish CDSMP)	8.9%	9.5%	8.9%	10.1%	7.0%
**Delivery site types**					
Senior center/AAA	29.2%	30.5%	27.4%	30.5%	28.3%
Healthcare organizations	21.1%	23.0%	20.5%	20.5%	20.5%
Residential facilities	17.6%	13.4%	18.5%	19.8%	18.8%
Community/multi-purpose facilities	9.9%	9.2%	10.2%	10.2%	10.0%
Faith-based organizations	8.4%	9.3%	8.9%	6.7%	8.7%
Educational institutions	2.3%	2.7%	2.1%	1.5%	2.8%
County health departments	1.3%	1.4%	1.3%	1.0%	1.4%
Tribal organizations	0.2%	0.2%	0.2%	0.1%	0.2%
Workplaces	0.5%	1.0%	0.6%	0.4%	0.2%
Other	9.5%	9.4%	10.2%	9.3%	9.1%
**Workshop language**					
English	89.6%	89.3%	90.0%	87.4%	91.8%
Spanish	10.4%	10.7%	10.0%	12.6%	8.2%
**Number of participants enrolled in workshop**	12.69 (± 4.18)	12.61 (±4.22)	12.78 (±4.17)	12.66 (±4.18)	12.71 (±4.14)
**Number of sessions attended**	4.38 (± 1.72)	4.36 (± 1.75)	4.38 (± 1.71)	4.40 (± 1.68)	4.37 (± 1.72)
**Successful completion (attend 4+ sessions)**					
No	25.1%	25.9%	25.3%	23.9%	25.4%
Yes	74.9%	74.1%	74.7%	76.1%	74.6%
**Delivery site**					
Metro	79.6%	78.6%	77.6%	82.6%	79.6%
Non-Metro	20.4%	21.4%	22.4%	17.4%	20.4%
**Percent of families below poverty**	1128 (±5.39)	10.76 (±4.11)	11.41 (±5.49)	11.48 (±15.67)	11.46 (±16.05)

Workshop delivery characteristics remained relatively consistent across increments of 25,000 participants reached, although general trends were observed for some variables. For example, after reaching the first 25,000 participants, it took shorter amounts of time to reach subsequent groups of 25,000 participants (i.e., 9.37 months to reach the first 25,000 participants and 5.37 months to reach the last 25,000 participants). As more participants were reached, larger proportions participated in DSMP workshops (i.e., 8.3% for the first 25,000 participants and 12.0% for the last 25,000 participants) and fewer participated in Spanish-language workshops (i.e., 10.7% for the first 25,000 participants and 8.2% for the last 25,000 participants). Additionally, workshops were increasingly delivered in ZIP Codes with higher percentages of families residing below the federal poverty line (i.e., an average of 10.76 families below poverty for the first 25,000 participants and 11.46 for the last 25,000 participants).

### CDSME program participant characteristics

Table 2 presents participant characteristics of the first 100,000 CDSME program participants enrolled in the intervention. On average, the first 100,000 CDSME program participants were 67.09 (±14.58) years of age; 12.0% were under age 50 years, 42.7% were aged 65–79 years, and 19.9% were aged 80 years and older. The majority of participants was female (77.9%), non-Hispanic (82.6%), and white (66.0%). Approximately 22% of participants were African American, 4.5% were Asian or Pacific Islander, 1.6% American Indian or Native Alaskan, and 6.2% “other” or multiple races. The majority of participants resided with other individuals (84.4%) and lived in metro areas (78.2%). On average, participants self-reported 2.20 (±1.71) chronic conditions; 39.6% reported three or more co-morbidities.

**Table 2:** **Sample characteristics by reach increments of 25,000 participants**.

	Total	Participant 1–25,000	Participant 25,001–50,000	Participant 50,001–75,000	Participant 75,001–100,000
**Age (average)**	67.09 (±14.58)	67.37 (±14.31)	66.67 (±14.71)	67.28 (±14.62)	67.05 (±14.66)
**Age group**
Under 50	12.0%	11.0%	12.7%	12.2%	12.2%
50–64	25.3%	26.0%	26.3%	24.2%	24.6%
65–79	42.7%	42.7%	41.6%	43.2%	43.4%
80+	19.9%	20.3%	19.5%	20.3%	19.7%
**Sex**
Male	22.1%	21.9%	23.2%	21.1%	22.1%
Female	77.9%	78.1%	76.8%	78.9%	77.9%
**Hispanic ethnicity**
No	82.6%	80.9%	83.3%	80.5%	85.6%
Yes	17.4%	19.1%	16.7%	19.5%	14.4%
**Race**
White	66.0%	69.2%	65.0%	63.9%	66.3%
African American	21.7%	19.3%	22.4%	22.7%	22.1%
Asian/Pacific Islander	4.5%	5.2%	4.8%	4.2%	4.1%
American Indian/Alaska native	1.6%	1.4%	1.6%	1.8%	1.5%
Other/multiple races	6.2%	5.0%	6.3%	7.4%	6.0%
**Number of chronic conditions (average)**	2.20 (±1.71)	1.96 (±1.63)	2.25 (±1.17)	2.28 (±1.74)	2.31 (±1.73)
**Number of chronic conditions**
0 Conditions	18.2%	22.5%	17.1%	17.0%	16.3%
1 Condition	20.9%	22.0%	21.0%	20.6%	19.8%
2 Condition	21.3%	21.2%	21.1%	20.9%	21.9%
3+ Condition	39.6%	34.3%	40.7%	41.5%	42.0%
**Live alone**
No	84.4%	78.1%	86.0%	85.0%	88.7%
Yes	15.6%	21.9%	14.0%	15.0%	11.3%
**Participant residence**
Metro	78.2%	77.8%	77.0%	80.9%	77.2%
Non-metro	21.8%	22.2%	23.0%	19.1%	22.8%

Generally, participant characteristics remained consistent across increments of 25,000 participants reached; however, trends were observed for some variables. For example, as more participants were reached by CDSME programs, more participants with chronic conditions were enrolled, with the number of participants enrolling with three or more chronic conditions increasing from 34.3% for the first 25,000 participants to 42.0% for the last 25,000 participants (i.e., participant 75,001–100,000). Additionally, as more participants were reached, the program enrolled smaller proportions of participants who lived alone (i.e., decreasing from 21.9% for the first 25,000 participants to 11.3% for the last 25,000 participants).

## Discussion

Self-management education has been recognized as a critical factor in empowering adults to improve their health and functioning (3). This study provides valuable dissemination and implementation insights into the nature and progression of the largest ever national roll out of CDSME programs (i.e., highly effective evidence-based programs designed to help middle-aged and older adults more effectively manage their chronic conditions). The aging services sector, in partnership with other healthcare, public health, community, and faith-based organizations, proved to be a coordinated, efficient, and diverse delivery system capable of rapidly reaching large numbers of older adults across the country. Exceeding programmatic goals of having 50,000 participants complete CDSME program workshops (12), over 100,000 participants were reached more quickly than in previous efforts (16). Further, with the exception of a predominant female participant population typically served with health promotion programs (16–18), participants were representative of the U.S. population and not just easy-to-reach subgroups.

The ability of this initiative to quickly reach 100,000 participants can be attributed to many factors. First, having each state set ambitious yet feasible and attainable goals (19) can help stimulate them to think differently about program planning, participant recruitment, and partnership development. Second, the stimulus money utilized in this initiative was essential for reaching this recruitment goal, but it was also leveraged by funds from other organizations with some non-traditional partners (e.g., healthcare partners), which fostered growth by adopting and promoting CDSME programs as an integral care practice. Third, capitalizing on the existing program delivery infrastructure established by previous AoA initiatives, the broad network of delivery and funding partners has resulted in widespread financing of CDSME programs by other government organizations. Fourth, workshops were available in many local communities largely because of the cooperation of the program developers to utilize and expand their training infrastructure (5). As seen in this initiative, the culmination of leveraging opportunities led to the rapid dissemination of CDSME programs by creating a highly collaborative community structure that accelerated the speed of scalability across the country to meet the needs of an increasingly diverse group of participants.

Past reports have shown CDSME programs have capacity to serve large numbers of heterogeneous adults via a growing network of delivery sites (8, 12, 20, 21). Success can be attributed, in part, to a community-driven delivery system that employed existing networks for recruiting participants of varied ages, race/ethnicity; disease status; geographic region; and socio-economic status (22). However, additional efforts are needed to help CDSME programs gain major penetration among the over 35 million older Americans estimated to have at least one chronic condition (23). As such, this study suggests several actions that can help make the dissemination of CDSME programs part of routine care.

First, we must further examine and strengthen referral systems to CDSME programs and the interconnectedness of the healthcare, public health, and aging services networks. Multi-institute funding initiatives that highly encourage/mandate multi-sectorial partnerships can set the stage for bridging such connections (11). Second, we need to embrace the paradigm shift in provider-patient communications that emphasizes the value of “informed and activated” patients working collaboratively with their prepared practice team (1). This theme, initially articulated in Wagner’s chronic care model, is being revisited with the recent movement toward patient-centered care (24). Third, we need to be aware of the constraints facing today’s healthcare providers in terms of shortened office visits and greater expectations for administrative paperwork (25). Thus, we recommend easy-to-employ methods and mechanisms (e.g., automated systems) to help health care providers know where evidence-based programs like CDSMP are offered. Also, guidelines are needed for identifying the types of patients who are best suited for specific programs (e.g., information about the pros and cons of generic self-care programs versus disease-specific programs). While clinicians and other allied health providers should be trained about these guidelines and referral processes, it is also important that program participants report back to their healthcare providers about their experiences and progress in such programs. Fourth, we must recognize that programmatic scalability needs to be paired with plans for achieving sustainability over time. Thus, we recommend that national, state, and local roll outs of evidence-based programs include sustainability planning as a core element. Successful sustainability plans are those that build upon and leverage existing resources, often employing champions for recognizing and promoting new models of care (26).

There are study limitations that must be acknowledged. While this national effort afforded large numbers of participants, specific data points were limited due to community concerns regarding burden. Additionally some data was missing due to local/regional constraints, and not necessarily individual refusal. Study data represented a “snapshot” of an ongoing evolving evaluation process at a particular time point. Underserved populations (e.g., African Americans) were overrepresented in this study because of the focus of the larger initiative to serve this subgroup of Americans. However, males were underrepresented in this study, as they traditionally are in evidence-based programs delivered through the aging services network (16–18). Despite these limitations inherent when using administrative records, we nevertheless believe this study represents a unique examination about how a national evidence-based dissemination rolls out over time, what infrastructure facilitates this type of grand-scale roll out, and what types of participants are reached.

Findings from this study capture the spread of CDSME programs during a national, government-funded roll out and show the ability of this intervention to rapidly reach a diverse set of participants using a well-coordinated delivery system. As of August 2014, over 196,700 participants reached by CDSME programs through 17,500 workshops in 1,200 counties across the United States. While this initiative capitalized and built upon previous efforts to create a delivery infrastructure for CDSME programs, this grand-scale dissemination has solidified the presence of CDSME programs with great potential for long-term sustainability. While this initiative has achieved impressive participant reach and completion, it should be noted that many other organizations throughout the United States offered the intervention despite not receiving this ARRA funding. Because data from these organizations are not represented in the databased used in the current study, these findings are even more encouraging in that they underrepresent the actual translation of CDSME programs nationwide. Continued efforts are needed to track the progression and proliferation of this suite of programs to empower patients with self-management skills to deal with their chronic conditions.

## Conflict of Interest Statement

The authors declare that the research was conducted in the absence of any commercial or financial relationships that could be construed as a potential conflict of interest.

This paper is included in the Research Topic, “Evidence-Based Programming for Older Adults.” This Research Topic received partial funding from multiple government and private organizations/agencies; however, the views, findings, and conclusions in these articles are those of the authors and do not necessarily represent the official position of these organizations/agencies. All papers published in the Research Topic received peer review from members of the Frontiers in Public Health (Public Health Education and Promotion section) panel of Review Editors. Because this Research Topic represents work closely associated with a nationwide evidence-based movement in the US, many of the authors and/or Review Editors may have worked together previously in some fashion. Review Editors were purposively selected based on their expertise with evaluation and/or evidence-based programming for older adults. Review Editors were independent of named authors on any given article published in this volume.
